# Stereotypic immune response in *Mycobacterium avium* ssp. *paratuberculosis* infection among different Swiss caprine genotypes

**DOI:** 10.1177/03009858251322726

**Published:** 2025-03-17

**Authors:** Melina Rasper-Hössinger, Simone Scherrer, Roger Stephan, Frauke Seehusen

**Affiliations:** 1 University of Zurich, Zurich, Switzerland

**Keywords:** *Mycobacterium avium* ssp. *paratuberculosis*, C-type, S-type, goats

## Abstract

Paratuberculosis is an infection with *Mycobacterium avium* ssp. *paratuberculosis* (MAP) causing chronic enteritis in domestic and wild ruminants worldwide. In goats, the infection is caused by C (cattle)-type and S (sheep)-type strains. In this study, the correlation between different MAP strains and histomorphological lesions in the small and large intestines, as well as the mesenteric lymph nodes, in Swiss goats (*Caprae aegagrus hircus*) was investigated. Ten Swiss caprine MAP isolates were characterized using polymerase chain reaction (PCR) and enzymatic restriction-based single nucleotide polymorphism (SNP) analysis. In addition, mycobacterial interspersed repetitive units and variable-number tandem repeats (MIRU-VNTR) profiling was performed, and the correlation with histologic lesions, scored as previously described for goats, was analyzed. Furthermore, immunohistochemical expression of CD3, CD79a, Iba1, cleaved caspase 3, and interleukin (IL)-17 was evaluated, and a morphometric analysis was conducted to quantify the different inflammatory cells. Diffuse multibacillary lesions were found in C-type/L’Institut national de recherche pour l’agriculture, l’alimentation et l’environnement (INRAE) Nouzilly MIRU-VNTR (INMV)1 (2/10) and S-type/INMV220 (1/10) animals. Diffuse lymphocytic lesions occurred in C-type/INMV1 (2/10) animals, while diffuse mixed lesions were observed in S-type/INMV218 (3/10) and S-type/INMV220 (2/10) animals. No significant differences in intestinal histological lesion scores were detected between S- and C-type INMV strains. Morphometrical analysis revealed similar inflammatory and apoptotic cell numbers in the intestinal mucosa of C- and S-type animals; however, S-type animals exhibited significantly more Iba1- and cleaved caspase 3-positive cells in mesenteric lymph nodes. Lesions in mesenteric lymph nodes might indicate a differentially regulated course in MAP pathogenesis.

Paratuberculosis, also known as Johne’s disease, is a widespread disease in cattle, sheep, goats, and other ruminant species caused by *Mycobacterium avium* subsp. *paratuberculosis* (MAP).^39^ This disease leads to substantial economic losses in the domestic livestock industry. Trading and introduction of subclinically infected animals are thought to be the most likely routes for MAP entry into ruminant herds, although wildlife reservoirs might also play a role in spreading the bacteria to livestock.^25^ The infection typically occurs during the first few months of life and young animals primarily become infected by exposure to feces from infected adult animals or their environment.^32^ In contrast to often clear clinical symptoms in cattle, which present as excessive watery diarrhea and weight loss, the disease exhibits a subtler progression in small ruminant flocks or herds. Affected animals eventually exhibit gradual weight loss, exercise intolerance, and soft feces in some cases, accompanied by chronic granulomatous enteritis and lymphadenitis.^41^

Based on their host species associations, MAP has been categorized into 2 main groups: the “sheep-type” (also known as “S-type” or “type I and III”) and the “cattle-type” (also referred to as “C-type” or “type II”).^4,30^ Goats infected with paratuberculosis are commonly associated with C-type MAP, but infections with S-type MAP are not uncommon and have been previously documented.^10,16^ In contrast to C-type strains, S-type strains exhibit slower growth and are more fastidious in culture media.^15,39,40^ Generally, C-type MAP demonstrates a higher infectivity and greater ability to survive within macrophages compared to S-type MAP.^29^

The histomorphology of paratuberculosis tissue lesions varies, and different classifications for lesions of the intestine and lymphatic tissue have been established for sheep,^3,21^ goats,^5^ and cattle.^14^ Lesions in naturally infected small ruminants have been categorized based on their location in relation to intestinal lymphoid tissue, severity of inflammatory lesions, types of inflammatory cells, and the number of acid-fast bacilli (AFB) present.^5,21^ Based on the histomorphology of the intestinal lesions, 2 primary forms have been described in animals showing clinical signs. The “paucibacillary” form is characterized by an inflammatory infiltrate composed mainly of lymphocytes and scattered macrophages with few to no intracellular AFB, and the “multibacillary” form, characterized by macrophages as the primary inflammatory infiltrate and numerous intracellular AFB.^3,5,21^ Furthermore, intestinal lesions consisting of focal small granulomas, exclusively in intestinal lymphoid tissues, were observed in small ruminants without clinical signs or grossly visible lesions.^5,21^

Besides macrophages, T and B lymphocytes, and plasma cells, other cell types may also play a role in the pathogenesis of caprine MAP infection.^35^ Several studies have shown that interleukin (IL)-17, which is primarily produced by Th17 cells, γδ T cells, and other innate immune cells, is involved in the pathogenesis of infectious and autoimmune diseases.^19,36^ After stimulation of caprine peripheral blood mononuclear cells with whole-cell sonicate of MAP, elevated IL-17 secretion was noted.^28^ In goats with orf, a virally induced cutaneous disease, elevated IL-17 levels point toward a critical role of IL-17-driven inflammation in the lesion development.^33^ Therefore, IL-17 might display a potential therapeutic target.^33^

Apoptosis, or programmed cell death, serves as a regulated mechanism for eliminating cells. Its significance lies not only in the removal of infected cells but also in maintaining immune system balance. Caspases, specific proteases dependent on cysteine and aspartate, are the primary enzymes orchestrating the apoptotic cascade, initiating and executing this process.^9^ Despite its importance, relatively little is known about the role of lymphocytic apoptosis in Johne’s disease.^13^ Studies examining gene expression reveal elevated levels of apoptosis-related genes in peripheral blood mononuclear cells from cattle with subclinical MAP infection compared to healthy animals.^8^ Conversely, goats with subclinical MAP infection display minimal apoptotic cells in lesions.^35^

In this study, a potential correlation between different MAP strains found in fecal samples of 10 goats and histological lesions of the intestine and lymph nodes from the same animals was investigated. This was accomplished by first examining and characterizing the different MAP strains by performing a specific single nucleotide polymorphism (SNP) analysis that distinguishes between C-type and S-type MAP, and a mycobacterial interspersed repetitive unit and variable-number tandem repeat (MIRU-VNTR) using 8 established loci. As a second step, the histological lesions of the small and large intestines and mesenteric lymph nodes, and the number of mycobacteria, were assessed and graded. Furthermore, a morphometrical analyses of the inflammatory infiltrate and cleaved caspase 3 (CC3)-positive cells in the intestine and mesenteric lymph nodes were performed.

## Material and Methods

### Animals and Samples

Ten adult goats from 4 Swiss farms with a clinical history of weight loss and chronic diarrhea were submitted for a postmortem examination at the owner’s request. A full postmortem examination was performed, and tissue and fecal samples were collected. No animal experiments were conducted as part of this study. In compliance with the local legislation, ethical approval was not required.

### DNA Extraction of Fecal Material and Tissue Samples

The extraction of genomic DNA from tissue (jejunum, ileum, colon, and mesenteric lymph node) and feces was performed as previously described.^26^ Tissue samples for DNA extraction were frozen at −20°C. A small piece of tissue (approximately 500 mg to 1 g) was cut from the thawed samples and incubated in 360 μl of ATL buffer (Qiagen, Hilden, Germany) for DNA extraction. For fecal samples, DNA was extracted by adding 1 ml ATL buffer (Qiagen) to 0.5 g of homogenized fecal sample. The samples were then transferred to a bead beating matrix in a 2-ml microtube (Omni International, Kennesaw, USA), heat inactivated at 99°C and subjected to mechanical cell lysis using the TissueLyser II (Qiagen). This was followed by enzymatic digestion with 40 μl proteinase K (Qiagen). Automated DNA preparation was carried out using 200 μl of the digested sample on the QIAcube instrument with the QIAamp cador Pathogen Mini Kit (Qiagen), following the manufacturer’s recommendations, with a DNA elution volume of 100 μl. The volume of eluted DNA corresponded to approximately 500 mg of tissue. DNA concentration was measured with a NanoDrop 2000c Spectrophotometer (Thermo Scientific, Reinach, Switzerland), and the extracts were stored at −20°C until use.

### Identification and Characterization of MAP

Each sample was investigated for the presence of the *F57* gene and IS*900* using an in-house quantitative polymerase chain reaction (qPCR).^27^ Samples were considered positive if both of the target genes, *F57* and IS*900*, had a C_t_-value of less than 38.

Differentiation between C-type and S-type strains was determined using an SNP assay based on PCR and restriction enzyme digestion of amplified PCR products, as previously described.^17,27^ Briefly, an enzymatic restriction assay using BsmBI on a PCR product involving SNP3842359 was performed in the first step. A resulting PCR product of 528 bp indicated the presence of C-type MAP, whereas 2 PCR fragments of 312 bp and 216 bp indicated the presence of S-type MAP. Only DNA samples that were clearly amplified in the PCR were included for further examination by MIRU-VNTR.

Genomic DNA was analyzed using eight established MIRU-VNTR targets.^34^ Each reaction mixture contained HotStart *Taq* Master Mix Kit (Qiagen), Q-Solution (Qiagen; only for loci VNTR 10 and VNTR 32), 0.5 µM of each primer, and 20 ng of purified genomic DNA in a final volume of 10 µl. PCR was performed for one cycle at 15 min at 95°C followed by 45 cycles at 95°C for 30 s, 60°C for 30 s, and 72°C for 30 s, and a final step at 72°C for 10 min. The PCR amplification product was analyzed by capillary electrophoresis (QIAxcel, Qiagen) using a high-resolution cartridge (Qiagen), a QX 15 bp–3 kb alignment marker (Qiagen), and a QX 100 bp–2.5 kb size marker (Qiagen). The assignment of the length of PCR products was performed using QIAxcel ScreenGel Software version 1.3.0 (Qiagen). As a positive control, reference strain MAP ATCC 19698 was tested in each PCR run. L’Institut national de recherche pour l’agriculture, l’alimentation et l’environnement (INRAE) Nouzilly MIRU-VNTR (INMV) profiles were determined according to a previously described allele-calling table (http://mac-inmv.tours.inra.fr/).

### Histological and Immunohistological Examination

The collected tissue samples were fixed in 10% buffered formalin and embedded in paraffin. Sections of 3 to 5 μm were prepared and stained with hematoxylin and eosin. For samples from the small and large intestines and mesenteric lymph nodes, consecutive sections were stained with Ziehl Neelsen stain for detection of AFB. The histological scores of intestinal lesions were assessed as previously described.^5,14,21^ In the small and large intestines and mesenteric lymph nodes, an immunohistochemical examination using antibodies directed against macrophages/monocytes (Iba1; Wako Chemicals, Neuss, Germany, Ref. 019-19741, dilution 1:750), T lymphocytes (CD3; Dako, Agilent Technologies, Glostrup, Denmark, clone F7.2.38, Ref. M725401, dilution 1:150), B lymphocytes (CD79a; Bio-Rad, clone HM57, Ref. MCA2538T, dilution 1:3000), apoptotic cells (CC3; Cell Signaling 9664L, Ref. P42574, dilution 1:400), and interleukin-17 producing cells (IL-17; HUABIO, Ref. ER1706-91, dilution 1:400) was performed as previously described.^26,27^ Briefly, after deparaffinization and rehydration, samples were subjected to antigen retrieval treatment (CD3, CD79a, and CC3: heat, pH buffer 9.0; Iba1 and IL-17: heat, pH buffer 6.0). For secondary antibodies anti-mouse (CD3 and CD79a) and anti-rabbit (Iba1, CC3, and IL-17) EnVision HRP system (code no. K4001/4003, Dako, Agilent Technologies, Glostrup, Denmark) were applied. Visualization was achieved by application of chromogen diaminobenzidine. Finally, slides were slightly counterstained with hemalaun and mounted. As negative controls, a replacement of the primary antibody with either a rabbit IgG (Abcam, Ref. ab37415) or mouse IgG isotype control (Abcam, Ref. ab37355) with the same protein concentration as the primary antibody was applied. Formalin-fixed and parrafin-embedded lymphoid tissues from healthy goats were used as positive controls for lymphocyte subsets, macrophages, and apoptotic cells.

### Morphometric Analysis

A morphometric approach was taken to quantify the percentage of macrophages, T-cells, B-cells, apoptotic cells, and IL-17 producing cells in the small and large intestine and mesenteric lymph node. Slides immunolabeled for Iba1, CD3, CD79a, IL-17, and CC3) were scanned using a digital slide scanner (NanoZoomer-XR C12000; Hamamatsu, Hamamatsu City, Japan) with an objective lens of 20× and a scanning resolution of 0.46 μm/pixel, followed by an evaluation with the computer program VIS (Visiopharm Integrator System, Version 5.0.4. 1382; Visiopharm, Hoersholm, Denmark). Sections immunolabeled for Iba1, CD3, CD79a, IL-17, and CC3 were used to quantify the total Iba1-, CD3-, CD79a-, IL-17-, and CC3-positive area in the tissue. For all quantitative approaches, one or several cross-sections of mesenteric lymph node and intestines were selected as regions of interest using a tissue detection app created within the Visiopharm software. This included the mucosa, submucosa, muscularis, and serosa of intestines as well as every aspect of the nodal tissue. A quantitative assessment was performed on the selected areas, and the means of the measurements of each organ were calculated and used for statistical analysis. A threshold classification method allowed recognition of the immunolabeled area, and the results were expressed as the immunolabeled area (in µm^2^) per total area of the region of interest (in µm^2^). In a postprocessing step, very small positive areas (<5 μm^2^) were excluded from counting to avoid falsely classifying areas of increased background labeling as cells. Regions of interest and immunolabeled areas were measured in µm^2^, then a percentage was calculated.

### Statistical Analysis

Data obtained by immunohistochemistry were subjected to statistical analysis using the software IBM SPSS Statistics software (version 29; IBM, Armonk, NY, USA) employing the Mann–Whitney U-test as group-wise test for comparison of C- and S-types, as the data were not normally distributed. A *P* value of <.05 was considered statistically significant. Box plots with integrated data points were created in *R* (R, version 4.2.2 [2022-10-31 ucrt], R Foundation for Statistical Computing, Vienna, Austria).

## Results

### Characterization of MAP Samples

MAP *F57* and IS*900* were successfully amplified for all 10 MAP samples, with C_t_-values ranging from 10.11 to 31.3 in the jejunum, 9.7 to 25.2 in the ileum, 16.7 to 29.05 in the colon, 16.94 to 28.9 in the mesenteric lymph node, and 15.81 to 25.32 in feces. Differentiation between C- and S-type MAP using an SNP assay revealed that 40% (n = 4) of samples belonged to C-type MAP and 60% (n = 6) belonged to S-type MAP. MIRU-VNTR profiling displayed the presence of “new” strains INMV218 (30%, n = 3) and INMV220 (30%, n = 3), as well as the “classic” strain INMV1 (40%, n = 4). Strains INMV218 and INMV220 were classified as S-type, while INMV1 was classified as C-type (Table 1).

**Table 1.** Total number of MAP samples classified into INMV groups and C- and S-type.

INMV Group^a^	Number of Tandem Repeats at MIRU-VNTR Loci^b^								C/S-Type	Number of Samples
	**292**	**X3**	**25**	**47**	**3**	**7**	**10**	**32**		
INMV 1	4	2	3	3	2	2	2	8	C	4
INMV 118	3	2	3	3	2	2	2	8	S	3
INMV 220	3	2	3	3	2	2	1	8	S	3

Abbreviations: MAP, *Mycobacterium avium* ssp. *paratuberculosis*; INMV, L’Institut national de recherche pour l’agriculture, l’alimentation et l’environnement (INRAE) Nouzilly MIRU-VNTR; C, cattle; S, sheep; MIRU-VNTR, mycobacterial interspersed repetitive units and variable-number tandem repeats.

a MIRU-VNTR tandem repeats classified into INMV groups.^32^

b Eight established MIRU-VNTR loci classified numerically according to the MAC INMV database (http://mac-inmv.tours.inra.fr/).

### Pathological Findings

In the course of postmortem examinations, various macroscopic findings were observed. The most common alterations were enlarged mesenteric lymph nodes (*n* = 7) and emaciation or a cachectic nutritional status (n = 6). Other observed lesions included watery content in the intestinal lumen, interpreted as diarrhea (n = 3), throracic or abdominal effusions (n = 3), peritonitis (n = 2), endoparasitosis (n = 2), and pseudotuberculosis (n = 1). No macroscopic findings were observed in the intestinal mucosa.

Paratuberculosis lesions in the small and large intestines, as well as the mesenteric lymph nodes, were histologically classified according to the previously described criteria for goats. This classification was based on the presence, location, intensity, and distribution of granulomatous lesions; the cell types present in the infiltrate; and the presence and number of AFB in the lesions of intestine and lymphatic tissue.^5^ Lesions associated with MAP infection were classified as described in the following paragraphs.

#### Diffuse multibacillary lesions

**Figure 1.**
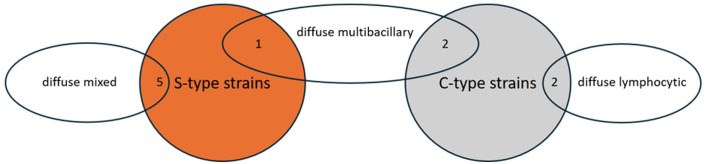
Distribution and number of histological lesion types associated with S-type (orange) or C-type strains (gray) of *Mycobacterium avium* ssp. *paratuberculosis*.

**Figure 2.**
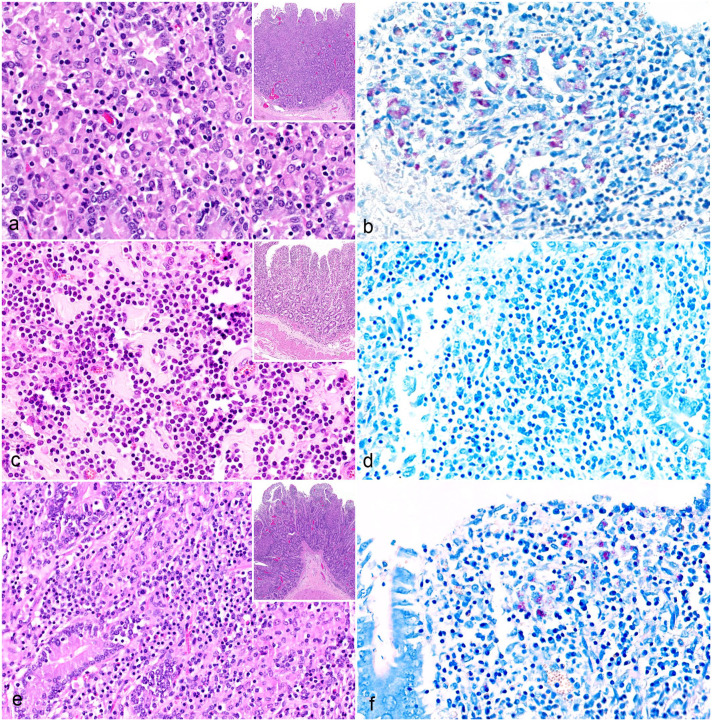
Paratuberculosis, small intestine, goat. (**a**) Diffuse multibacillary lesion. Infiltration of the lamina propria with mainly macrophages, fewer lymphocytes, and plasma cells. Inset: overview, small intestine. Case 3. Hematoxylin and eosin (HE). (**b**) Diffuse multibacillary lesion. Numerous intracytoplasmic acid-fast bacilli (AFB) in macrophages. Case 3. Ziehl-Neelsen (ZN) acid-fast stain. (**c**) Diffuse lymphocytic lesion. Infiltration of the lamina propria with mainly lymphocytes, fewer macrophages, and plasma cells. Inset: overview, small intestine. Case 6. HE. (**d**) Diffuse lymphocytic lesion. No AFB visible with a ZN stain. Case 6. (**e**) Diffuse mixed lesion. Infiltration of the lamina propria with a mixed inflammatory infiltrate consisting of macrophages and lymphocytes. Inset: overview, small intestine. Case 4. HE. (**f**) Diffuse mixed lesion. Moderate numbers of intracytoplasmic AFB in macrophages. Case 4. ZN.

Diffuse multibacillary lesions were seen in 3 goats (cases 3, 5, and 9), representing C- (cases 3 and 5) and S-type MAP (case 9; Fig. 1, Table 2). In these cases, the mucosa of the jejunum and ileum was moderately thickened due to granulomatous enteritis, characterized by diffuse infiltration of the lamina propria by macrophages, often with abundant eosinophilic, partially granulated to foamy cytoplasm (epithelioid macrophages). A low to moderate number of lymphocytes and few plasma cells were evenly distributed among the epithelioid macrophages (Fig. 2a). Multinucleated giant cells were detected multifocally. The Ziehl-Neelsen stain revealed moderate to high numbers of AFB within the cytoplasm of the epithelioid macrophages, mainly in the superficial mucosa of jejunum and ileum (Fig. 2b). In the Peyer’s patches, a multifocal to coalescing infiltration of macrophages was visible. All animals exhibited a low to moderate number of intracytoplasmic AFB in macrophages within the Peyer’s patches.

**Table 2.** Classification of histological lesions and MAP samples classified into INMV groups and C- and S-type MAP strains with the corresponding goats (case 1-10).

Histologic Classification	Animal	C/S-Type	INMV Group^a^
Diffuse multibacillary	3	C	1
	5	C	1
	9	S	220
Diffuse lymphocytic	6	C	1
	10	C	1
Diffuse mixed	1	S	218
	2	S	218
	4	S	218
	7	S	220
	8	S	220

Abbreviations: MAP, *Mycobacterium avium* ssp. *paratuberculosis*; INMV, L’Institut national de recherche pour l’agriculture, l’alimentation et l’environnement (INRAE) Nouzilly MIRU-VNTR; C, cattle; S, sheep; MIRU-VNTR, mycobacterial interspersed repetitive units and variable-number tandem repeats.

a MIRU-VNTR tandem repeats classified into INMV groups.^32^

The submucosa of all goats was moderately thickened by an infiltrate consisting of mainly macrophages, lymphocytes, fewer plasma cells, and transparent to pale-eosinophilic homogenous material (edema). Furthermore, various degrees of lymphangiectasia were visible. Multifocally in the lamina muscularis and serosa of cases 3 and 5, low to moderate numbers of lymphocytes and macrophages, few plasma cells, mild edema, and lymphangiectasia were visible, often in a perivascular distribution.

In the large intestine of cases 5 and 9, there was mild to moderate, diffuse infiltration of the lamina propria and submucosa by lymphocytes, plasma cells, and nonepithelioid macrophages with a mild to moderate number of intracytoplasmic AFB, consistent with a chronic lymphoplasmacytic colitis. The investigated mesenteric lymph nodes were also infiltrated by the epithelioid macrophages. The Ziehl-Neelsen stain revealed a low to moderate number of AFB in the mesenteric lymph nodes of cases 3 and 9.

#### Diffuse lymphocytic lesions

Diffuse lymphocytic lesions were observed in 2 goats (cases 6 and 10), which were infected with C-type MAP (Fig. 1, Table 2). Lesions consisted of a severe, diffuse lymphohistiocytic enteritis, with lymphocytes as the predominant inflammatory cells infiltrating the lamina propria of the jejunum and ileum. Among them, a moderate number of macrophages and few plasma cells were present (Fig. 2c). Multinucleated giant cells were rarely detected. In the Peyer’s patches, there was multifocal to coalescing infiltration of macrophages. The Ziehl-Neelsen stain revealed no AFB in the mucosa of the jejunum, ileum, or Peyer’s patches (Fig. 2d).

The submucosa of both goats had mild, multifocal infiltration of lymphocytes, macrophages, fewer plasma cells, eosinophilic granulocytes, and edema. Various degrees of lymphangiectasia were also visible. No histological lesions were detected in the lamina muscularis and serosa.

Similar to the multibacillary form, the large intestine of all goats contained a mild diffuse infiltrate in the lamina propria mucosae and submucosa that was composed of lymphocytes, plasma cells, and nonepithelioid macrophages, consistent with a mild to moderate chronic lymphoplasmacytic colitis. The mesenteric lymph nodes contained a multifocal to coalescing infiltrate primarily composed of macrophages. No AFB were detected in the large intestine and only a small number of intracytoplasmic AFB were present in macrophages in the mesenteric lymph node of case 10.

#### Diffuse mixed lesions

Diffuse mixed lesions were found in five goats (cases 1, 2, 4, 7, and 8), which were all infected with S-type MAP (Fig. 1, Table 2). Lesions in these goats were characterized by a diffuse granulomatous enteritis, composed of a mixed infiltrate consisting of non-epithelioid macrophages, lymphocytes, and few plasma cells (Fig. 2e). Multinucleated giant cells were rarely detected. The infiltrate caused mild to moderate thickening of the mucosa and submucosa of jejunum and ileum. Variations in the cellular composition of the infiltrate within samples from the same animal were sometimes observed, with either macrophages or lymphocytes being more prominent. The Ziehl-Neelsen stain revealed moderate to high numbers of AFB within the cytoplasm of macrophages in the superficial mucosa of the jejunum and ileum of all goats (Fig. 2F). In the Peyer’s patches, there was multifocal to coalescing infiltration of non-epithelioid macrophages, but no intracytoplasmic AFB were visible.

In the submucosa of all goats, a mild to moderate multifocal to coalescing infiltrate, mainly non-epithelioid macrophages, lymphocytes, fewer plasma cells, and transparent to pale-eosinophilic homogenous material (edema), was visible. Furthermore, mild multifocal lymphangiectasia was observed. As in the lymphocytic form, no histological lesions were detected in the lamina muscularis and serosa.

The large intestine of all animals had mild diffuse infiltration of the lamina propria mucosae and submucosa by lymphocytes, plasma cells, and non-epithelioid macrophages, similar to the findings in the multibacillary and lymphocytic forms. The mesenteric lymph nodes contained a multifocal to coalescing infiltrate composed of mainly non-epithelioid macrophages. No AFB were detected in the large intestine or mesenteric lymph nodes.

### Morphometrical Analysis

#### Iba1-positive area in intestines and lymph nodes

**Figure 3.**
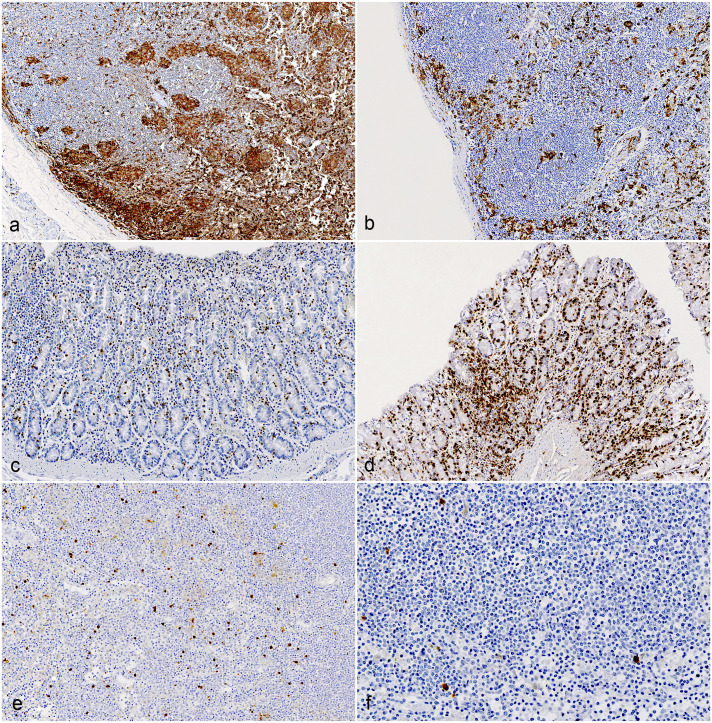
Comparison of immunohistochemistry (IHC) in mesenteric lymph nodes and colon of goats infected with C- and S-type *Mycobacterium avium* ssp*. paratuberculosis* (MAP). (**a**) Goats infected with S-type MAP show a higher percentage (*P* = 0.038) of Iba1-positive area in the mesenteric lymph node. (**b**) Goats infected with C-type MAP showed a significantly lower percentage of Iba1-positive area in the mesenteric lymph node. (**c**) Goats infected with S-type MAP showed a significantly lower percentage (*P* = 0.024) of CD3-positive in the colonic mucosa. (**d**) Goats infected with C-type MAP showed a significantly higher percentage of CD3-positive area in the colonic mucosa. (**e**) Goats infected with S-type MAP showed a higher percentage (*P* = 0.019) of cleaved caspase 3 (CC3)-positive area in the mesenteric lymph node. (**f**) Goats infected with C-type MAP showed a significantly lower percentage of CC3-positive area in the mesenteric lymph node.

**Figure 4.**
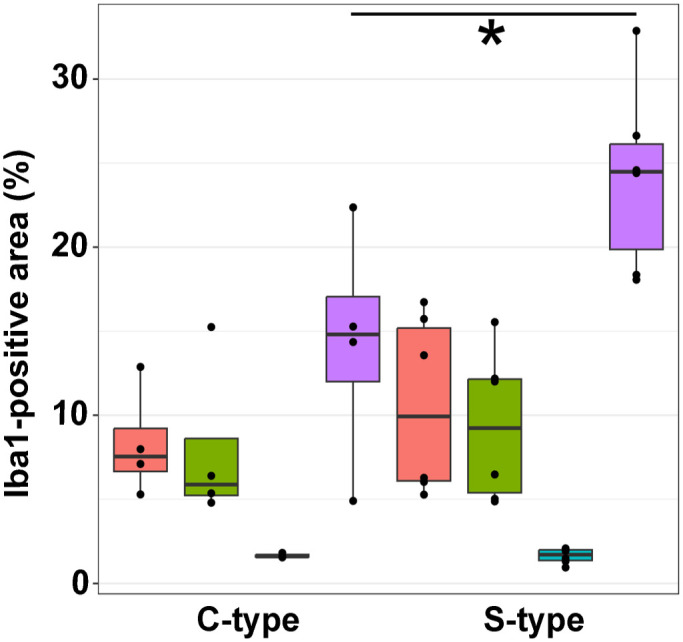
Comparison of Iba1-positive area (%) of the jejunum, ileum, colon, and mesenteric lymph node of goats infected with C- and S-type *Mycobacterium avium* ssp*. paratuberculosis*. Percentage of Iba1-positive area is significantly higher (*P* = 0.038) in mesenteric lymph nodes of goats infected with S-type MAP. Mann–Whitney U test, significant differences between groups are marked by brackets. Red = jejunum, green = ileum, blue = colon, violet = lymph node.

Immunoreactivity for Iba1 in the jejunum ranged from 5.29 to 16.74% of the total area across different groups, while in the ileum, it ranged from 4.8 to 15.55%. In the large intestine, immunolabeling ranged from 0.95 to 2.09%. Notably, the mesenteric lymph nodes showed labeling ranging from 4.91 to 32.88% across different groups. No significant differences were observed between C-type and S-type MAP infections in the small and large intestines; however, goats infected with S-type MAP exhibited significantly larger areas (*P* = 0.038) of Iba1-positive cells in the mesenteric lymph nodes (Figs. 3a, b and 4).

#### CD3-positive area in intestines and lymph nodes

**Figure 5.**
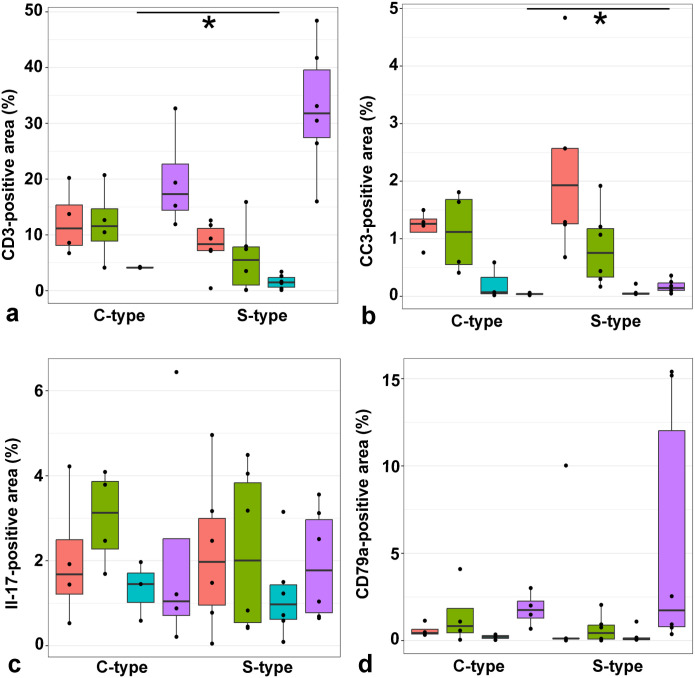
Comparison of CD3-, cleaved caspase 3 (CC3)-, IL-17-, and CD79a-positive area (%) of the jejunum, ileum, colon, and mesenteric lymph node of goats infected with C- and S-type *Mycobacterium avium* ssp*. paratuberculosis* (MAP). (**a**) Percentage of CD3-positive area is significantly higher (*P* = 0.024) in colons of goats infected with C-type MAP. (**b**) Percentage of CC3-positive area is significantly higher (*P* = 0.019) in mesenteric lymph nodes of goats infected with S-type MAP. (**c**) No significant differences between MAP types are detected in IL-17 immunohistochemistry. (**d**) No significant difference between MAP types were detected in CD79a immunohistochemistry. Mann–Whitney U test, significant differences between groups are marked by brackets. Red = jejunum, green = ileum, blue = colon, violet = lymph node.

In the jejunum, CD3 immunolabeling ranged from 0.43 to 20.23% of the total area across different groups, while in the ileum, it ranged from 0.11 to 20.72%. In the large intestine, immunolabeling ranged from 0.11 to 4.27%. Notably, the mesenteric lymph nodes showed labeling ranging from 11.92 to 48.42% across different groups. When comparing C-type and S-type MAP, goats infected with C-type MAP exhibited significantly larger areas (*P* = 0.024) of CD3-positive cells in the large intestine compared to those infected with S-type MAP; however, no significant differences were observed between C-type and S-type infections in the small intestine and mesenteric lymph nodes (Figs. 3c, d and 5a).

#### CC3-positive area in intestines and lymph nodes

Immunoreactivity for CC3 in the jejunum ranged from 0.68 to 4.84% of the total area across different groups, while in the ileum, it ranged from 0.17 to 1.92%. In the large intestine, immunolabeling ranged from 0.02 to 0.59%. Notably, the mesenteric lymph nodes showed labeling ranging from 0.02 to 0.36% across different groups. No significant differences were observed between C-type and S-type MAP infections in the small and large intestines; however, goats infected with S-type MAP exhibited significantly larger areas (*P* = 0.019) of CC3-positive cells in the mesenteric lymph nodes (Figs. 3e, f and 5B).

#### IL-17 positive area in intestines and lymph nodes

**Figure 6.**
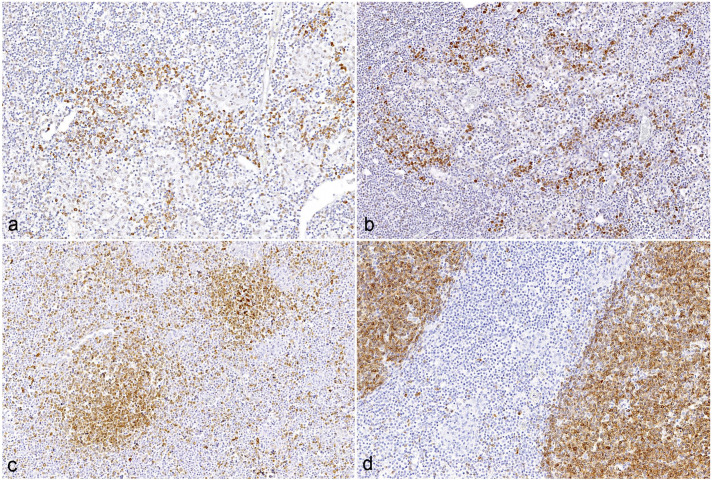
IL-17 and CD79a immunohistochemistry in mesenteric lymph nodes of goats infected with C- and S-type *Mycobacterium avium* ssp *paratuberculosis* (MAP). No significant differences in IL-17- and CD79a- positive area between MAP-types was detected (**a**) IL-17 immunolabeling in a goat infected with S-type MAP. (**b**) IL-17 immunolabeling in a goat infected with C-type MAP. (**c**) CD79a immunolabeling in a goat infected with S-type MAP. (**d**) CD79a immunolabeling in a goat infected with C-type MAP.

In the jejunum, IL-17 immunoreactivity ranged from 0.05 to 4.96% of the total area across different groups, while in the ileum, it ranged from 0.42 to 4.49%. In the large intestine, immunolabeling ranged from 0.09 to 3.15%, and in the mesenteric lymph nodes, it ranged from 0.21 to 6.44%. No significant differences in the IL-17-positive area were observed between C-type and S-type MAP infections across any of the examined tissues (Figs. 5c and 6a, b).

#### CD79a-positive area in intestines and lymph nodes

In the jejunum, CD79a immunoreactivity ranged from 0.01 to 10.03% of the total area across different groups, while in the ileum, it ranged from 0.05 to 4.10%. In the large intestine, immunolabeling ranged from 0.05 to 1.09%, and in the mesenteric lymph nodes, it ranged from 0.37 to 15.4% across different groups. Interestingly, no significant differences in the CD79a-positive area were observed between C-type and S-type MAP infections in any of the examined tissues (Figs. 5d and 6c, d).

## Discussion

In this study, a comparative approach was used to analyze different caprine MAP genotypes of naturally infected Swiss goats and their respective pathomorphological lesions. It is worth noting, that the number of animals in this study was quite limited, as the determination of genotypes by MIRU-VNTR typing and SNP analysis had to be accompanied by post-mortem examination for gross examination, followed by tissue collection and histological examination of samples from the same animal. This study design, therefore, differs from the usual practice of conducting large-scale MAP tests to rule out this notifiable animal disease in Switzerland, which typically involves testing fecal samples for MAP using PCR.

In our study, four animals exhibited an infection with C-type MAP, all of which could be assigned to the classic, well-known genotype INMV1.^2,6,23^ Among six goats, S-type MAP was detected, with 3 each assigned to the more recently detected genotypes INMV218^27^ and INMV220. Various phenotypic features linked to potential virulence factors differ noticeably between C-type MAP and S-type MAP. These differences include growth rate in culture,^4^ cell invasion, cytokine induction, persistence in macrophages, and pathological responses, including clinical manifestation, pathological lesions, immune responses, and bacterial shedding dynamics in experimentally infected lambs.^38^ In the study involving experimentally infected lambs, animals with C-type MAP histologically showed focal lesions, primarily located in the lymph nodes. These lesions were characterized by small granulomas with significant numbers of giant cells. In lambs infected with S-type MAP, histological lesions appeared mainly in the intestine and had a multifocal or, more frequently, a diffuse and severe character without the presence of giant cells.^38^ However, in our study, goats infected with C-type and S-type MAP did not exhibit this specific distribution of histological lesions in intestine and mesenteric lymph nodes. Goats infected with both C-type and S-type MAP showed diffuse lesions in small intestines and mesenteric lymph nodes, where giant cells were frequently visible in diffuse multibacillary and mixed types, and no discernible differences in the composition of lymphocytic subtypes and the numbers of macrophages, apoptotic cells, and IL-17 producing cells within the mucosa of the small intestine were found between C- and S-genotypes in the morphometric analysis. However, significant differences in the mesenteric lymph nodes were observed between C-type and S-type MAP. Goats infected with the S-type MAP exhibited significantly higher numbers of macrophages and apoptotic cells compared to those infected with the C-type. In addition, the presence of diffuse mixed lesions exclusively in S-type MAP-infected animals may indicate a differential immune response between the MAP types.

Goats can be infected with both C- and S-type MAP strains. However, in naturally infected goats, MAP isolates predominantly belong to the C-type strains, with S-type strains being comparatively rare.^4,31^ Moreover, when C-type strains were inoculated into goats either as *in vivo* isolates or as cultured bacteria, they demonstrated significantly greater pathogenicity compared to both *in vivo* and *in vitro* S-type strains. This increased pathogenicity was manifested by higher rates of shedding and clinical disease, as well as stronger interferon-gamma and antibody responses, particularly with bovine MAP strains.^31^ In the current investigation of naturally infected goats, 60% of the MAP isolates were identified as S-type. Interestingly, no differences were observed in the extent of clinical symptoms and macroscopic changes between the isolates. These results are comparable to a study in cattle, in which no differences were found between MAP isolates regarding macroscopic lesions in the intestinal mucosa.^20^ In a study involving naturally infected cattle, differences among the MAP isolates and the host response were observed. Results indicated that the most frequently found MAP-type (haplotype A) was associated with more severe histopathological lesions and a stronger immune response compared to the grouped nondominant MAP strains.^37^

Besides a formerly suggested repression of gene expression in peripheral blood mononuclear cells of MAP-infected cows, IL-17 secretion was elevated *in vitro* in caprine and bovine peripheral blood mononuclear cells after stimulation with MAP antigen, supporting the importance of Th17 cells and IL-17 in the immune response to MAP.^7,28^ However, their exact role in infection control and disease development remains unclear.^11,12^ Th17 cells and IL-17 cytokines may play important roles in the immune response to MAP infection, contributing to inflammation and immune modulation.^12^ However, prolonged Th17 activation may exacerbate disease progression.^12^ Our results indicate no difference in the IL-17-mediated immune response elicited by C- and S-type MAP. Further investigation is warranted to understand the implications of this finding in the context of MAP infection and disease progression.

Previous studies have shown conflicting results regarding apoptosis in MAP-infected macrophages. In an *in vitro* study, it was observed that macrophages infected with bovine C-type MAP strains contained a lower proportion of spontaneously apoptotic cells compared to uninfected cell populations. In addition, these infected cells exhibited significantly reduced activation of caspases and lower levels of *caspase 3*, *7*, and *8* mRNA, which may account for their diminished capacity to undergo apoptosis.^16^ Conversely, other *in vitro* studies have reported that MAP can induce cell death in macrophages^1,22^ and that apoptosis is dependent on the MAP burden, meaning apoptosis was not observed in infections with a low burden of MAP, whereas both apoptosis and necrosis were observed in infections with a high burden of MAP.^22^ Similar results were found in a study with naturally infected cattle where diffuse multibacillary lesions, which exhibit higher levels of MAP, showed larger areas for CC3-positive cells than diffuse intermediate lesions.^18^ In our study, no differences were observed in apoptotic, CC3-positive cells among different histological lesion types in the intestine. Multibacillary lesions, which showed higher levels of AFB compared to mixed and lymphocytic lesions, did not display a significantly higher number of apoptotic cells. In partial concordance to our results, a gene expression study in white blood cells of subclinically infected sheep did not identify apoptosis as a differentially regulated pathway.^24^ In a study comparing lymphocyte apoptosis in peripheral blood mononuclear cells and lymph node cells from sheep experimentally exposed to MAP and from healthy, non-exposed sheep, apoptosis in intestinal lymph node cells increased in response to MAP antigen in MAP-infected sheep, but not in MAP-exposed uninfected sheep, indicating a MAP-mediated lymphocyte apoptosis during disease progression, which possibly contributes to the immune dysfunction in Johne’s disease.^13^ However, in our study, goats infected with S-type MAP exhibited a significantly higher number of apoptotic, CC3-positive cells and Iba1-positive cells in the mesenteric lymph node compared to animals infected with C-type MAP, possibly indicating a different immune response in lymphoid organs. In calves experimentally infected with MAP, lymph nodes were persistently infected early in the disease course, with cytokine expression levels indicating a Th1 immune response. This response, characterized by the production of cytokines like interferon-gamma, suggests an active attempt by the immune system to combat the MAP infection.^42^

Further research is needed to understand the differential pathogenicity of C-type and S-type MAP strains. In addition, gaining a better understanding of the role of Th17 cells and IL-17 cytokines in the immune response to MAP infection could provide valuable insights for disease control and management strategies.

## References

[1] Bannantine JP Stabel JR. Killing of *Mycobacterium avium* subspecies *paratuberculosis* within macrophages. BMC Microbiol. 2002;2(1):1–7. 11860602 10.1186/1471-2180-2-2 PMC65544

[2] Bryant JM Thibault VC Smith DGE , et al. Phylogenomic exploration of the relationships between strains of *Mycobacterium avium* subspecies paratuberculosis. BMC Genomics. 2016;17(1):1–12. 10.1186/s12864-015-2234-5 PMC4729121 26813574

[3] Carrigan MJ Seaman JT. The pathology of Johne’s disease in sheep. Aust Vet J. 1990;67(2):47–50. 2344336 10.1111/j.1751-0813.1990.tb07693.x

[4] Collins DM Gabric DM De Lisle GW. Identification of two groups of *Mycobacterium paratuberculosis* strains by restriction endonuclease analysis and DNA hybridization. J Clin Microbiol. 1990;28(7):1591–1596. 2166089 10.1128/jcm.28.7.1591-1596.1990 PMC267994

[5] Corpa JM Garrido J García Marín JF , et al. Classification of lesions observed in natural cases of paratuberculosis in goats. J Comp Pathol. 2000;122(4):255–265. 10805979 10.1053/jcpa.1999.0368

[6] Correa-Valencia NM Moyano RD Hernández-Agudelo M , et al. *Mycobacterium avium* subsp. *paratuberculosis* (MAP) molecular diversity in cattle, sheep, and goats from Latin America and the Caribbean: a systematic review. Trop Anim Health Prod. 2021;53(5):1–11. 10.1007/s11250-021-02923-9 PMC8453475 34546430

[7] Coussens PM Colvin CJ Wiersma K , et al. Gene expression profiling of peripheral blood mononuclear cells from cattle infected with Mycobacterium paratuberculosis. Infect Immun. 2002;70(10):5494–5502. 12228275 10.1128/IAI.70.10.5494-5502.2002 PMC128350

[8] Coussens PM Pudrith CB Skovgaard K , et al. Johne’s disease in cattle is associated with enhanced expression of genes encoding IL-5, GATA-3, tissue inhibitors of matrix metalloproteinases 1 and 2, and factors promoting apoptosis in peripheral blood mononuclear cells. Vet Immunol Immunopathol. 2005;105(3–4):221–234. 15808302 10.1016/j.vetimm.2005.02.009

[9] Creagh EM Conroy H Martin SJ. Caspase-activation pathways in apoptosis and immunity. Immunol Rev. 2003;193(1):10–21. 12752666 10.1034/j.1600-065x.2003.00048.x

[10] de Juan L Álvarez J Aranaz A , et al. Molecular epidemiology of Types I/III strains of *Mycobacterium avium* subspecies *paratuberculosis* isolated from goats and cattle. Vet Microbiol. 2006;115(1–3):102–110. 16500045 10.1016/j.vetmic.2006.01.008

[11] DeKuiper JL Cooperider HE Lubben N , et al. *Mycobacterium avium* subspecies *paratuberculosis* drives an innate Th17-like T cell response regardless of the presence of antigen-presenting cells. Front Vet Sci. 2020;7:108. 10.3389/fvets.2020.00108 PMC7089878 32258066

[12] DeKuiper JL Coussens PM. Inflammatory Th17 responses to infection with *Mycobacterium avium* subspecies *paratuberculosis* (MAP) in cattle and their potential role in development of Johne’s disease. Vet Immunol Immunopathol. 2019;218:109954 31733610 10.1016/j.vetimm.2019.109954

[13] De Silva K Browne S Begg DJ , et al. Apoptosis of lymph node and peripheral blood cells in ovine Johne’s disease. Vet Immunol Immunopathol. 2013;156:82–90. 24054092 10.1016/j.vetimm.2013.08.001

[14] González J Geijo MV García—Pariente C , et al. Histopathological classification of lesions associated with natural paratuberculosis infection in cattle. J Comp Pathol. 2005;133(2–3):184–196. 16045917 10.1016/j.jcpa.2005.04.007

[15] Juste RA Marco á JCS iez De Oc á iriz C , et al. Comparison of different media for the isolation of small ruminant strains of Mycobacterium paratuberculosis. Vet Microbiol. 1991;28(4):385–390. 1949552 10.1016/0378-1135(91)90073-o

[16] Kabara E Coussens PM. Infection of primary bovine macrophages with *Mycobacterium avium* subspecies *paratuberculosis* suppresses host cell apoptosis. Front Microbiol. 2012;3:215 22833736 10.3389/fmicb.2012.00215 PMC3400940

[17] Leão C Goldstone RJ Bryant J , et al. Novel single nucleotide polymorphism-based assay for genotyping *Mycobacterium avium* subsp. paratuberculosis. J Clin Microbiol. 2016;54(3):556. 26677250 10.1128/JCM.01958-15 PMC4767949

[18] Lucena AN Garza-Cuartero L McAloon C , et al. Apoptosis levels in bovine Johne’s disease ileal lesions and association with bacterial numbers. Vet Pathol. 2021;58(6):1086–1090. 34190009 10.1177/03009858211025790 PMC8581713

[19] Matsuzaki G Umemura M. Interleukin-17 family cytokines in protective immunity against infections: role of hematopoietic cell-derived and non-hematopoietic cell-derived interleukin-17s. Microbiol Immunol. 2018;62(1):1–13. 29205464 10.1111/1348-0421.12560

[20] Möbius P Liebler-Tenorio E Hölzer M , et al. Evaluation of associations between genotypes of *Mycobacterium avium* subsp. *paratuberculosis* and presence of intestinal lesions characteristic of paratuberculosis. Vet Microbiol. 2017;201:188–194. 28284609 10.1016/j.vetmic.2017.01.026

[21] Perez V Marin JFG Badiola JJ. Description and classification of different types of lesion associated with natural *paratuberculosis* infection in sheep. J Comp Pathol. 1996;114(2):107–122. 8920212 10.1016/s0021-9975(96)80001-6

[22] Periasamy S Tripathi BN Singh N. Mechanisms of *Mycobacterium avium* subsp. *paratuberculosis* induced apoptosis and necrosis in bovine macrophages. Vet Microbiol. 2013;165(3–4):392–401. 23639474 10.1016/j.vetmic.2013.03.030

[23] Pickrodt C Köhler H Moog U , et al. Molecular diversity of *Mycobacterium avium* subsp. paratuberculosis in four dairy goat herds from Thuringia (Germany). Animals. 2023;13(22):3542. 38003160 10.3390/ani13223542 PMC10668697

[24] Purdie AC Plain KM Begg DJ , et al. Gene expression profiles during subclinical *Mycobacterium avium* subspecies *paratuberculosis* infection in sheep can predict disease outcome. Sci Rep. 2019;9(1):1–15. 31160677 10.1038/s41598-019-44670-w PMC6547741

[25] Raizman EA Wells SJ Jordan PA , et al. *Mycobacterium avium* subsp. *paratuberculosis* from free-ranging deer and rabbits surrounding Minnesota dairy herds. Can J Vet Res. 2005;69(1):32. 15745220 PMC1142167

[26] Rasper-Hössinger M Biggel M Stephan R , et al. Strain diversity in *Mycobacterium avium* subsp. *paratuberculosis*-positive bovine fecal samples collected in Switzerland. Front Vet Sci. 2023;10:1154516. 37180063 10.3389/fvets.2023.1154516 PMC10171428

[27] Scherrer S Stephan R Zumthor JP , et al. *Morphological and molecular characterization* of a new *Mycobacterium avium* subsp. paratuberculosis S-type strain genotype in goats. Front Vet Sci. 2019;6:250. 31417916 10.3389/fvets.2019.00250 PMC6684744

[28] Stabel JR Bannantine JP Hostetter JM. Comparison of sheep, goats, and calves as infection models for *Mycobacterium avium* subsp. paratuberculosis. Vet Immunol Immunopathol. 2020;225:110060. 32413513 10.1016/j.vetimm.2020.110060

[29] Stevenson K . Genetic diversity of *Mycobacterium avium* subspecies *paratuberculosis* and the influence of strain type on infection and pathogenesis: a review. Vet Res. 2015;46(1):64. 26092160 10.1186/s13567-015-0203-2 PMC4473831

[30] Stevenson K Hughes VM De Juan L , et al. Molecular characterization of pigmented and nonpigmented isolates of *Mycobacterium avium* subsp. paratuberculosis. J Clin Microbiol. 2002;40(5):1798–1804. 11980962 10.1128/JCM.40.5.1798-1804.2002 PMC130953

[31] Stewart DJ Vaughan JA Stiles PL , et al. A long-term study in Angora goats experimentally infected with *Mycobacterium avium* subsp. *paratuberculosis*: clinical disease, faecal culture and immunological studies. Vet Microbiol. 2006;113(1-2):13–24. 16310981 10.1016/j.vetmic.2005.09.015

[32] Sweeney RW. Transmission of paratuberculosis. Vet Clin North Am Food Anim Pract. 1996;12(2):305–312. 8828107 10.1016/s0749-0720(15)30408-4

[33] Tang X Jing T Chen X , et al. Interleukin-17 mediates inflammatory tissue injury during orf development in goats. Vet Microbiol. 2021;258:109105. 33991787 10.1016/j.vetmic.2021.109105

[34] Thibault VC Grayon M Boschiroli ML , et al. New variable-number tandem-repeat markers for typing *Mycobacterium avium* subsp. *paratuberculosis* and M. avium strains: comparison with IS900 and IS1245 restriction fragment length polymorphism typing. J Clin Microbiol. 2007;45(8):2404–2410. 17537942 10.1128/JCM.00476-07 PMC1951273

[35] Valheim M Sigurðardóttir G Storset A , et al. Characterization of macrophages and occurrence of T cells in intestinal lesions of subclinical paratuberculosis in goats. J Comp Pathol. 2004;131(2-3):221–232. 15276862 10.1016/j.jcpa.2004.04.004

[36] Vantourout P Hayday A. Six-of-the-best: unique contributions of γδ T cells to immunology. Nat Rev Immunol. 2013;13(2):88–100. 23348415 10.1038/nri3384 PMC3951794

[37] Verdugo C Marquez D Paredes E , et al. Association between the severity of histopathological lesions and *Mycobacterium avium* subspecies *paratuberculosis* (MAP) molecular diversity in cattle in southern Chile. Front Vet Sci. 2023;9:962241 36713883 10.3389/fvets.2022.962241 PMC9878319

[38] Verna AE Garcia-Pariente C Muñoz M , et al. Variation in the immuno-pathological responses of lambs after experimental infection with different strains of Mycobacterium avium subsp. Paratuberculosis. Zoonoses Public Health. 2007;54(6-7):243–252. 17803513 10.1111/j.1863-2378.2007.01058.x

[39] Whittington RJ Hope AF Marshall DJ , et al. Molecular epidemiology of *Mycobacterium avium* subsp. *paratuberculosis*: IS900 restriction fragment length polymorphism and IS1311 polymorphism analyses of isolates from animals and a human in Australia. J Clin Microbiol. 2000;38(9):3240–3248. 10970365 10.1128/jcm.38.9.3240-3248.2000 PMC87366

[40] Whittington RJ Marsh IB Saunders V , et al. Culture phenotypes of genomically and geographically diverse *Mycobacterium avium* subsp. *paratuberculosis* isolates from different hosts. J Clin Microbiol. 2011;49(5):1822. 21430104 10.1128/JCM.00210-11 PMC3122651

[41] Windsor PA . Managing control programs for ovine caseous lymphadenitis and paratuberculosis in Australia, and the need for persistent vaccination. Vet Med (Auckl). 2014;5:11–22. 32670842 10.2147/VMRR.S44814 PMC7337174

[42] Wu CW Livesey M Schmoller SK , et al. Invasion and persistence of *Mycobacterium avium* subsp. *paratuberculosis* during early stages of Johne’s disease in calves. Infect Immun. 2007;75(5):2110–2119. 17296749 10.1128/IAI.01739-06 PMC1865790

